# Identifying Potential Candidate Hub Genes and Functionally Enriched Pathways in the Immune Responses to Quadrivalent Inactivated Influenza Vaccines in the Elderly Through Co-Expression Network Analysis

**DOI:** 10.3389/fimmu.2020.603337

**Published:** 2020-12-04

**Authors:** Jing Yang, Jiayou Zhang, Renfeng Fan, Wei Zhao, Tian Han, Kai Duan, Xinguo Li, Peiyu Zeng, Jinglong Deng, Jikai Zhang, Xiaoming Yang

**Affiliations:** ^1^ National Institute of Engineering Technology Research in Combination Vaccine, Wuhan, China; ^2^ Wuhan Institute of Biological Products Co., Ltd., Wuhan, China; ^3^ Guangdong Province Institute of Biological Products and Materia Medica, Guangzhou, China; ^4^ Gaozhou Center for Disease Control and Prevention, Maoming City, China; ^5^ China Biotechnology Co., Ltd., Peking, China

**Keywords:** weighted gene co-expression network analysis (WGCNA), hub genes, quadrivalent inactivated influenza vaccines (QIVs), *MCEMP1*, *SPARC*, reactogenicity

## Abstract

Insights into the potential candidate hub genes may facilitate the generation of safe and effective immunity against seasonal influenza as well as the development of personalized influenza vaccines for the elderly at high risk of influenza virus infection. This study aimed to identify the potential hub genes related to the immune induction process of the 2018/19 seasonal quadrivalent inactivated influenza vaccines (QIVs) in the elderly ≥60 years by using weighted gene co-expression network analysis (WGCNA). From 63 whole blood samples from16 elderly individuals, a total of 13,345 genes were obtained and divided into eight co-expression modules, with two modules being significantly correlated with vaccine-induced immune responses. After functional enrichment analysis, genes under GO terms of vaccine-associated immunity were used to construct the sub-network for identification and functional validation of hub genes. *MCEMP1* and *SPARC* were confirmed as the hub genes with an obvious effect on QIVs-induced immunity. The *MCEMP1* expression was shown to be negatively correlated with the QIVs-associated reactogenicity within 7 days after vaccination, which could be suppressed by the CXCL 8/IL-8 and exacerbated by the Granzyme-B cytotoxic mediator. Meanwhile, the *SPARC* expression was found to increase the immune responses to the QIVs and contribute to the persistence of protective humoral antibody titers. These two genes can be used to predict QIVs-induced adverse reaction, the intensity of immune responses, and the persistence of humoral antibody against influenza. This work has shed light on further research on the development of personalized QIVs with appropriate immune responses and long-lasting immunity against the forthcoming seasonal influenza.

## Introduction

In the industrialized countries, influenza associated deaths usually occur in adults ≥ 65 yrs (years) (1). Based on the estimation model of influenza-related mortality ratios, the risk of death associated with seasonal influenza is higher in the age group ≥65 yrs than in the age group <65 yrs annually, resulting in a tremendous disease burden in the regions with more aging populations (2). Studies have shown that immunosenescence may decrease the capacity of the immune system and increase influenza associated morbidity and mortality (3, 4). Although influenza vaccination can protect against infection and its complications, as well as reduce the risk of hospitalization and the severity of influenza-related diseases, the effectiveness and efficacy of seasonal influenza vaccines varied with vaccination coverage, season, sex, health status, race/ethnicity and age (5, 6). The critical factor of aging immunity contributed to a lower cell-mediated immune response to the influenza vaccination in the elderly individuals > 65 yrs than in those < 65 yrs, leading to the failure to offer sufficient protection against seasonal circulating influenza virus (7–10), with an over 31% decrease observed in the vaccine effectiveness in the elderly individuals aged 65 yrs or above in the two A(H3N2)-dominated influenza seasons (11), due to the poorly adapted B cell responses (12) and a rapid decline in the antibodies induced by the trivalent seasonal influenza vaccine (13, 14). China has entered an aging society, with 18.1% of its population or 253.88 million individuals aged 60 yrs and above as of January 2020, suggesting the necessity to understand the transcriptomic mechanism of immunity induction by the quadrivalent influenza vaccine in the elderly individuals and explore the potential hub genes and signaling pathways for the development of effective quadrivalent seasonal influenza vaccines (15).

Several systems vaccinology studies have been performed in humans to elucidate the immunogenicity of seasonal influenza vaccines from multidimensional levels in adults, identifying early IFN signatures at the transcriptome level (16) and inducing positive memory B cells through early activation of circulating memory T follicular helper cells(ICOS+CXCR3+CXCR5+CD4+ T cells, Tfh) (17), which contributed to a higher antibody response and long-lasting humoral immunity, respectively. This novel systems vaccinology approach focused on the interaction between vaccines and the entire host systems (18). Systems vaccinology provides a powerful tool to analyze a large amount of biological data from omics technology to reconstruct the biological processes contributing to the success or failure of the influenza vaccines for the elderly and establish a standard influenza vaccine prototype.

To obtain critical immune-related information from the intricate mechanisms related to the immune responses to influenza vaccines, a powerful analytical method is needed to mine vaccine-induced immunity information from large omics datasets. Convenient and inexpensive access to human peripheral blood for accurate diagnosis and monitoring of post-immunization conditions suggests the need to identify reliable biomarkers from peripheral blood to assess the effectiveness and safety of QIVs in the elderly after inoculation. Several valuable biomarkers were identified by using next-generation sequencing, such as *GCN2* (general control nonderepressible 2 kinase), with a strong correlation between its expression and the enhancement of antigen presentation (19). It is a tough challenge to identify one single gene signature or biomarker to understand or predict vaccine response through paired sample analysis of differentially expressed genes. As the subjects with baseline heterogeneity at the transcriptome level, this limitation is inevitable, resulting in the loss of useful information, low abundance or no statistical fold-change. Peripheral whole blood contains various types of immune cells, which can provide a large amount of RNA expression data, but require effective analytical methods to compress these high-dimensional gene expression data into a few modules of eigengenes with a close functional relationship.

Weighted Gene Co-expression Network Analysis (WGCNA) is commonly used to explore both weighted and un-weighted gene correlation networks from RNA-Seq data, such as a hierarchical clustering analytical method to measure the functional relationship between genes or modules (20). This computational methodology can be used to reconstruct a complete picture of vaccine-induced immune responses from a network-focused perspective and provide systematic insights without losing any information in paired sample analysis. WGCNA has been effectively used to identify disease-associated co-expression modules and eigengenes, and interpret large amounts of RNA-Seq data as biological process information (21, 22). To our best knowledge, few studies have been performed by using this methodology to investigate the vaccine-induced immunity, compare, or verify the results of previous systems vaccinology studies in seasonal influenza vaccination.

The purpose of this study was to identify the potential candidate hub genes and functionally enriched pathways in the immune responses of the elderly to QIVs by WGCNA analysis of the RNA-Seq data sampled from the elderly individuals aged ≥60 yrs after one dosage of QIV vaccination. The dynamic and kinetic changes of transcriptional RNA expression and humoral antibody titer in these individuals were monitored by collecting venous blood samples at four time points pre- and post-vaccination as the immunological processes induced by influenza vaccines. Finally, 8 modules were constructed by WGCNA with the progression of QIV immunization to identify the potential hub genes and functionally enriched pathways.

## Materials and Methods

The study population and laboratory detection protocols described below are based on those published in previous studies (23, 24).

### Recruitment

Briefly, a total of 1920 medically healthy subjects were enrolled into a randomized, double-blind, controlled phase III, and non-inferiority clinical trial in the southeast of China [Registration Numbers: CTR20190846; subjects ≥ 60 yrs; perturbation: the 2018/19 seasonal quadrivalent inactivated influenza split vaccines (QIVs)]. From this enrolled population, 60 elderly participants were randomly selected for cell-mediated immunity analysis, and 16 of them with distinct demographic characteristics and HAI titers were chosen to identify the intricate mechanisms related to various immune phenotypes by multidimensional analyses, such as whole blood transcriptome and plasma cytokine expression.

The 1920 medically healthy recipients were immunized intramuscularly in the deltoid muscle of the nondominant arm with one dosage of the 2018/19 seasonal quadrivalent inactivated influenza split vaccines {QIVs, the experiment vaccine by Wuhan Institute of Biological Products Co., Ltd., lot: SH201805649; the control vaccine by Hualan Biological Engineering Co., Ltd., lot: 201809B033; containing the A/Michigan/45/2015 NYMC X-275A [**H1N1**; an A/Michigan/45/2015(H1N1)pdm09 like virus], A/Singapore/INFIMH-16-0019/2016 IVR-186 [**H3N2**; an A/Singapore/INFIMH-16-0019/2016(H3N2) like virus], B/Phuket/3073/2013 wild type virus [**B Yamagata lineage**], and B/Colorado/06/2017 wild-type virus [**B Victoria lineage**]}. Both the chicken egg-derived QIVs adopted the same vaccine production process with standard dosage formulation of 15 µg per virus strain.

The whole blood samples (~ 4 ml each) of the 60 subjects were promptly collected at day 0 (pre-vaccination), 3, 28, and 180 post-vaccination in conventional vacuum tubes with EDTA-K2 anticoagulant by professional phlebotomists in Gaozhou Center for Disease Control and Prevention, where the clinical study was conducted (Gaozhou, Guangdong, China).

### Hemagglutination Inhibition (HAI) Assay

Serum samples from subjects inoculated with QIVs were obtained pre-vaccination and 28 days after immunization. Hemagglutination-inhibiting (HAI) assay was applicated for immunogenicity of the vaccine against vaccine homologous formulations.

The HAI assay applied in accordance with standard WHO procedures (25) to assess immune responses post-vaccination. A 1% suspension of chicken erythrocytes and 4 HA units/25 μl of corresponding influenza virus antigens were employed in the detection of functional antibody titers. Serum samples were treated with receptor destroying enzyme and tested in duplicate in serial 2-fold dilutions initiating from 1:10. And each test plate contained both negative and positive serum controls. The HI titer was defined as the inverse of the highest serum dilution to inhibit hemagglutination. For the convenience of calculation and statistical analysis, HI antibody titers below 10 were treated as 5.HI antibody titer ≥40 was defined as seroprotection/seropositive, which was deemed to provide humoral immune protection against specific influenza viruses.

### Ethics Statement

Before this clinical study, a written informed consent was obtained from each subject. The clinical protocols were approved by the Clinic Institutional Ethics Review Board of Guangdong Centers for Disease Control and Prevention, and the study was performed according to the local institutional ethics committee guidelines. The trial was registered with China Drug Trials.org.cn (Registration Numbers: CTR20190846; subjects ≥ 60 years).

### Data Availability Statement

RNA-Seq Expression data in our study were deposited at the National Center for Biotechnology Information Gene Expression Omnibus (GEO) public repository (accession number: GSE151558), to review GEO accession GSE151558 at https://www.ncbi.nlm.nih.gov/geo/query/acc.cgi?acc=GSE151558.

## Statistical Methods

### mRNA Next-Generation-Sequencing (Illumina HiSeq Platform)

The mRNA sequencing was performed as previously reported (26, 27). Briefly, total RNA was extracted from each EDTA-K2 anticoagulant supplemented with whole blood sample using TRIzol reagent (Bio Basic Inc., Canada) according to the manufacturer’s instructions, followed by constructing the sequencing libraries and isolating poly-A RNA from total RNA by Poly-T oligo-attached magnetic beads (Invitrogen, CA, USA). Paired-end read sequencing was performed on an Illumina Hiseq platform (Illumina NovaSeq 6000), and 125/150 bp paired-end reads were generated. Hisat2 v2.0.5 was used to build the index of the reference genome (hg19) and align the paired-end clean reads to the reference genome. The variability in RNA-seq data was removed with the Conditional Quantile Normalization (CQN) method (28). The number of reads mapped in the reference genome was counted by FeatureCounts version (1.5.0-p3). Subsequent analyses were based on these normalized values, including 18,849 genes with counts over 0 at one of these four time points (Days 0, 3, 28, and 180).

### Weighted Gene Co-expression Network Analysis (WGCNA)

The WGCNA algorithm was used to construct the hierarchical clusters containing genes with similar or identical functions using the standard gene expression data obtained at the four time points (Days 0, 3, 28, and 180) as previously reported (29). Prior to similarity matrix construction, all pairwise genes were calculated for Pearson’s correlation $\left(S=|S_{ij}|=|\frac{1+cor(i,j)}{2}|\right)$ to show the similarity between gene expression profiles, followed by transforming the similarity matrix into an adjacency matrix using the soft power adjacency function approach $\left(a_{ij}=power\left(S_{ij},\beta \right)\equiv {\left|S_{ij}\right|}^{\beta }\right)$, which can exhibit the potential factorization superiority and maintain the integrity of omics information. The optimal parameter *β* (the soft threshold *β*=2 was chosen in this analysis, with a minimum module size of 30 genes) was chosen to satisfy the scale-free topology appropriately. The topological overlap matrix (TOM= *ω_ij_*) was converted into a dissimilarity measure by the following equation $\left(d_{ij}^{\omega }=1-\omega_{ij},\omega_{ij}=\frac{lij+aij}{\min\{ki,kj\}+1-aij}\right)$ based on the adjacency function parameter, and the TOM-based dissimilarity measure and average linkage hierarchical clusters were applied in the definition of gene modules. Each module of eigengenes represented the function of a module, which was used to generate the correlation coefficients between modules and merge the highly correlated modules (a Pearson correlation coefficient of over 0.75). Finally, the Spearman correlation between these clustering eigengenes and the immune status of the subjects was calculated to reveal the underlying mechanisms involved in vaccine-induced immunity and immunosenescence of the elderly individuals ≥ 60 yrs as well as the related hub genes and functionally enriched pathways.

### Analysis of Module Interactions and the Association of Health Status With the Modules of Interest in the Elderly

To investigate the correlation of the divided co-expression modules, the eigengenes of each corresponding module were used for interaction analysis and heat map visualization. The main objective of this co-expression network analysis was to establish the relationship between these co-expression modules and the immune status of the elderly and identify the hub genes contributing to the modular connectivity and functionality. The traits of immune status in the elderly included gender, age, medical history, basal body temperature, general reaction, body mass index (BMI), blood pressure differential, and seroprotection against the four influenza vaccine strains (H1N1, H3N2, B_Y_ and B_V_), and the correlation between co-expression modules and these traits was calculated. The co-expression modules of a significant correlation to the traits related to influenza vaccine-induced immunity were defined as playing a critical role in the process of effective active immunization with QIVs. Besides, genes with messy expression in the grey module of high correlation with these traits may contribute to diagnose the immunogenicity of QIVs in the elderly. The subsequent analysis focused on the gene co-expression modules of the highest correlation to the traits described above.

### Gene Enrichment Analysis of Clustering Genes

The co-expression genes in the modules of interest were extracted for subsequent gene enrichment and network analyses to identify the genes involved in important biological regulation and signaling pathways contributing to efficient influenza vaccine-induced immune responses (30). Cluster Profiler R package was used to test the statistical enrichment of genes in Gene Ontology (GO) and Kyoto Encyclopedia of Genes and Genomes (KEGG) pathways (http://www.genome.jp/kegg/), where adjusted *P*-value < 0.05 was considered significantly enriched and the top 10 significant terms were visualized.

### Differential Expression Analysis of Immune Status-Related Genes in Clusters of Interest

Differentially expressed gene analysis was employed in the expression profiles of critical genes among the older adults with variant immune responses to QIVs, using the DESeq2 R package (1.16.1) based on the approach of Benjamini and Hochberg, with cut-off criterion set as FDR (false discovery rate, *p*adj) <0.05 and |log_2_fold change| >1 (31).

### Identification of Hub Genes

The hub genes in modules with higher correlation to the subject traits and efficient immune responses to the influenza vaccination may contribute to understanding the underlying transcriptional mechanisms of influenza vaccine response in the elderly. After GO enrichment analysis of the subject traits-correlated modules, the hub genes were excavated from the divided clusters with significant enrichment in immunity-related GO terms by constructing the sub-network. Cytoscape version 3.7.2 software was applied for the gene sub-network visualization based on its plug-in cytohubba function (32, 33), and the genes with high Maximal Clique Centrality (MCC) values were defined as hub genes. Subsequently, the hub genes in each corresponding module were excavated and imported into the STRING database (Version: 11.0) to obtain sub-network from the six protein-protein interaction (PPI) sources (Textmining, Experiments, Databases, Co−expression, Neighborhood, Gene Fusion) with a high confidence of not less than 0.4 and disconnected nodes hidden in the network for exploring Protein-Protein Interactions. Gene significance (GS) was denoted as correlation of gene expression profile with one subject trait, while the transcript module membership (MM) score was calculated by correlating the module eigengene with intramodular corresponding gene transcript expression. These associated functions and the index of screened hub genes could add weight to the identification robustness of indispensable regulatory impacts of interested hub genes on the immunity induced by influenza vaccines. For each trait in the elderly, the driver genes of corresponding modules were screened out by the highest absolute values of GS and MM generated by WGCNA.

### Quantitative Real-Time PCR (qRT-PCR)

Briefly, total RNA was extracted from whole blood sample using combined protocol with TRIzol and Takara MiniBest universal RNA extraction kit (Takara Bio Inc., Japan). Using PrimeScript™ RT Master Mix (Perfect Real Time) (Takara Bio Inc., Japan) to synthesize cDNA from 250 ng of total RNA in each sample, according to the manufacturer’s protocol. The housekeeping gene *GAPDH* (glyceraldehyde-3-phosphate dehydrogenase) was employed as internal reference. The primers of two genes were designed as follows: *MCEMP1* forward, 5’-*AGCCATCCTGAGCCTGTA*-3’ and *MCEMP1* reverse, 5’-*TGCCTGCTAATGTCGTCT*-3’; *SPARC* forward, 5’-*CAGAACCACCACTGCAAACA*-3’ and *SPARC* reverse, 5’- *AAGTGGCAGGAAGAGTCGAA*-3’. The SYBR Green Real time PCR Master Mix (Takara Bio Inc., Japan) was used to perform the qRT-PCR detection according to the manufacturer’s instructions and previous study (34). Each sample was detected in triplicate and the data was displayed as Mean FC (Fold Change) value and the Standard Error (SEM) relative to the expression of *GAPDH* gene based on 2^−ΔΔCT^ method (35).

### Plasma Cytokine Quantification

The concentrations of plasma cytokines (CCL4, Granzyme B, HGF, IFN-alpha, IFN-beta, IFN-gamma, CXCL 8/IL-8, IL-10, etc.) were detected using the xMAP technology according to the manufacturer’s instructions (LXSAHM-20, R&D Systems Inc.). Briefly, plasma samples were incubated for 2 h with antibodies conjugated to microspheres at room temperature, followed by incubation for 1 h with biotinylated antibodies and then 30 min with streptavidin-phycoerythrin fluorescent conjugate (SA-PE). The Luminex^®^ 200TM instrument (Luminex Corp.) was used to detect the signal intensity for each microsphere added to the protein samples. In this study, we only focused on two cytokines (CXCL 8/IL-8 and Granzyme-B) to demonstrate the dynamic status of pro-inflammation and cell-mediated immunity pre- and post-vaccination in the elderly.

Statistical analyses were conducted using IBM SPSS Statistics 26.0.0.0 (SPSS Software). A least significant difference (LSD) *post hoc*-test in one-way ANOVA was employed to compare multiple groups, with a 95% confidence interval.

## Results

### Demographic Characteristics and Traits of Immune Status

1920 medically stable adults aged 60 years and older were enrolled into the clinical study. Sixty subjects randomly selected from this clinical trial, with no significant medically healthy differences pre-vaccination (**Table 1**). The immunogenicity results could meet the European criteria (Committee for Medicinal Products for Human Use (CHMP) guidelines) against the four vaccine strains (H1N1, N3N2, B_YAM_ strain, and B_VIC_ strain) at day 28 after immunization (**Table 2**). Specifically, the Seroprotection Rates (SPRs) against H1N1, N3N2, B_VIC_, and B_YAM_ strain ranged in 94.38%–98.28%, the Seroconversion Rates (SCRs) against these four strains ranged in 74.14%–87.93%, and the Geometric Mean Increase (GMI) of antibody against these four strains increased 6.01–18.25 folds. The ranged existing immunity against influenza virus among the older subjects before vaccination, not only exhibited a history of influenza vaccination and/or infection, but also highlighted the individual variability in immune system. Subsequently, 16 subjects demonstrated distinct immune response phenotypes, were chosen for the integration analysis of the correlation of various phenotypes with transcriptional RNA expression.

**Table 1:** Demographic characteristics of the sixty randomly selected subjects.

Age Group	Age(yrs)	Age (Min, Max)(yrs)*	Number of Subjects(missing)	Sex Ratio (Male: Female)	Temperature Pre-Vaccination (Min, Max) (°C)*	Height (Min, Max) (cm)*	Weight (Min, Max) (kg)*
Older Adults	≥60	67.00 ± 5.10 (60.00, 80.00)	60 (0)	34:26	36.30 ± 0.30 (35.70, 36.90)	158.60 ± 9.20 (134.00, 179.00)	58.10 ± 9.08 (34.00, 80.00)

*The data were shown as Mean ± SD; yrs, years old; the number of subjects indicate those vaccinated with QIVs for the Full Analysis Set (FAS).

**Table 2:** The serological test results of QIVs against the vaccine strains (H1N1, N3N2, B_YAM_ strain, and B_VIC_ strain) by hemagglutinin inhibition assay (HAI).

Influenza vaccine virus strain	Indicators	Estimate [95%CI] at D0	Estimate [95%CI] at D28
		Older Adults (n=58)	Older Adults (n=58)
H1N1	SPR n (%)	22(37.93) [25.06–50.80]	55(94.38) [88.95–100.0]
	SCR n (%)	**/**	51(87.93) [79.29–96.57]
	GMT(1: )	18.39 [13.19–25.66]	335.69 [234.70–480.08]
	GMI	**/**	18.25 [12.92–25.78]
H3N2	SPR n (%)	17(29.31) [17.24–41.38]	56(96.55) [91.71–100.0]
	SCR n (%)	**/**	43(74.14) [62.52–85.75]
	GMT(1: )	18.18 [13.51–24.45]	135.35 [105.99–172.85]
	GMI	**/**	7.45 [5.30–10.45]
B_YAM_	SPR n (%)	48(82.76) [72.74–92.78]	57(98.28) [94.82–100.0]
	SCR n (%)	**/**	49(84.48) [74.88–94.09]
	GMT(1: )	52.65 [43.16–64.24]	316.20 [259.51–385.20]
	GMI	**/**	6.01 [4.63–7.79]
B_VIC_	SPR n (%)	18(31.03) [18.76–43.31]	55(94.83) [88.95–100.0]
	SCR n (%)	**/**	49(84.48) [74.88–94.09]
	GMT(1: )	19.53 [15.49–24.61]	178.17 [137.13–231.49]
	GMI	**/**	9.12 [6.95–11.98]

CI, conﬁdence interval; SPR, Seroprotection rates; SCR, Seroconversion rates; GMT, the geometric mean titer; GMI, the geometric mean increase of antibody titer; Seroprotection rates were deﬁned as the percentage of participants with an HAI titer≥40; Seroconversion rates were deﬁned as percentage of participants with either a pre-vaccination HAI titer <1:10 and a postvaccination HAI titer>1:40 or a pre-vaccination titer ≥1:10 and a ≥4-fold increase in post-vaccination titer.

The median age and age range of the 60 subjects selected were 67 yrs and 60–80 yrs, respectively, with female accounting for 43.3%. The detailed traits of immune status among the 16 older adults were listed in the **Table S1**. The pre-existing antibody titer against B_YAM_ strain was higher than that against the other three vaccine strains (H1N1, H3N2, B_VIC_) in the older adults.

### Data Preprocessing

Prior to the construction of hierarchical clusters, 18,849 genes were selected after normalization of raw gene counts, excluding the genes with no expression in all samples. A total of 64 samples were collected from 16 of the 60 subjects at four time points pre- and post-immunization, with one sample not collected at the fourth time point, thus 63 samples for further WGCNA clustering analysis.

### Construction of Weighted Gene Co-Expression Network and Correlation of the Co-Expression Modules With Health Status and Immunity Induced by Influenza Vaccines

**Figure 1:**
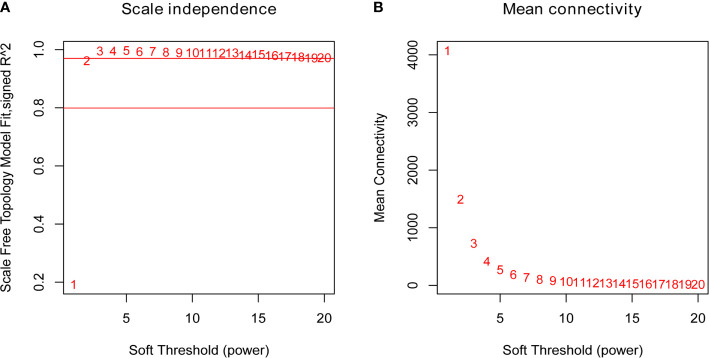
Plots of the Scale Free Topology Fitting Index **(A)** and the Mean Connectivity **(B)**. Each point was marked by the corresponding soft thresholding parameter *β*. *β*=2 was chosen as it could satisfy the criteria of a high R^2^ and a high mean number of connections.

**Figure 2:**
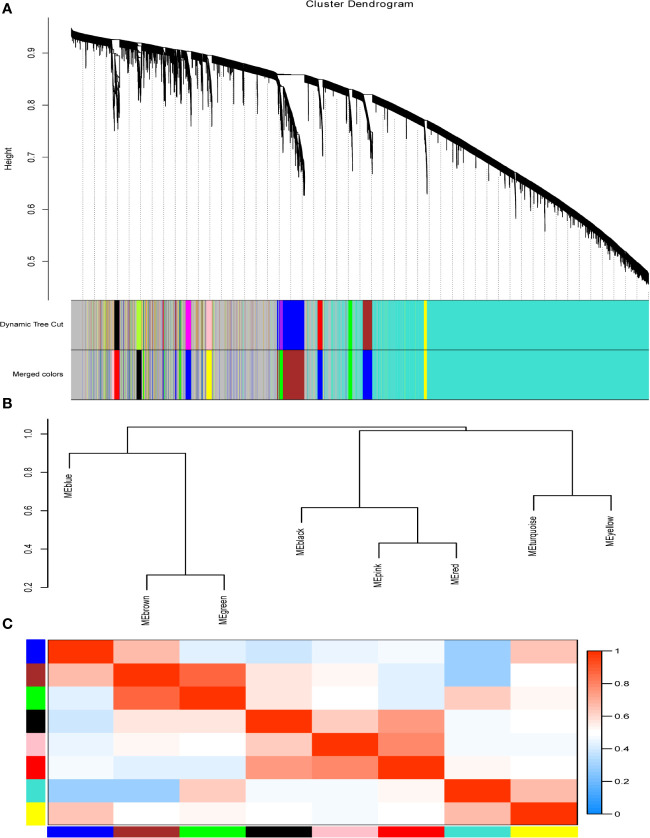
Modules for the progression of QIVs-induced immunity in the elderly. **(A)** Cluster Dendrogram of transcriptional expression genes with top 50% variance generated by using the TOM-based dissimilarity measure calculated as d ijω=1−ωij. The modules were labeled with different colors below the cluster dendrogram; the “Dynamic Tree Cut” row denoted the original clustering division and the ‘Merged Colors’ row displayed the final modules after merging the high eigengenes-based correlation coefficient modules. **(B)** Dendrogram of eigengenes in corresponding modules calculated using *P*earson’s correlation. **(C)** Adjacency heatmap of co-expression modules of eigengenes. Red represents high adjacency (positive correlation) and blue represents low adjacency (negative correlation). The meta-modules are along the diagonal.

To identify the hub genes which may regulate the immune responses to the QIVs in the elderly subjects, the similarly expressed genes in these samples were clustered into eight co-expression modules and marked with corresponding module colors, while the other genes were grouped into the grey module. Using the soft thresholding power (*β*=2) in this algorithm, the co-expression network could satisfy the approximate scale-free topology criterion with R^2^ > 0.80 while maintaining a high mean connectivity with enough information (**Figure 1**). We merged the modules of eigengenes with a correlation coefficient of over 0.75 (**Figure 2**), and the size of the co-expression modules ranged from 82 to 9,579 genes.

After merging the highly correlated modules, the co-expression genes were clustered into eight modules with similar gene expression patterns and labeled with different colors (**Figure 2A**), including the modules of Pink (82 genes), Black (163 genes), Red (173 genes), Green (487 genes), Yellow (613 genes), Brown (951 genes), Blue (1297 genes), and Turquoise (9579 genes), respectively. Grey module contained 5,504 genes that did not have the similar expression pattern with any modules. Genes within the corresponding modules indicated higher correlation than the genes between different modules from the network heatmap plot of selected genes (**Figure S1**). After visualizing the correlation of these eight modules, two clusters were yielded and each cluster could be further divided into two sub-clusters as displayed in (**Figures 2B, C** **)**.

**Figure 3:**
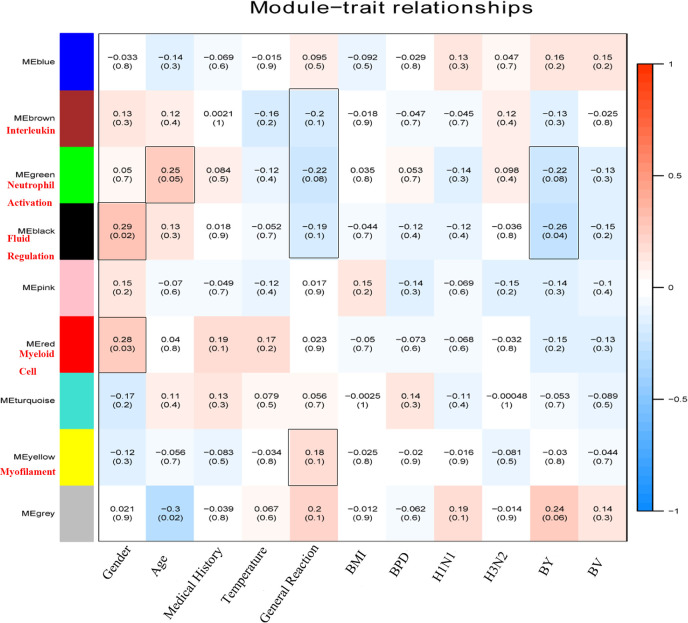
Heatmap plot of module-trait relationships. The correlation coefficients of module eigengenes with different health status and QIVs-induced immunity were calculated to identify the potential biological functions of each module. Each cell displayed the corresponding correlation coefficient and (*P-*value), and the cells with *P* ≤ 0.1 were highlighted in the box. The color depth indicated the intensity of correlation by the color legend (right), with red representing the positive correlation and blue representing the negative correlation. The gene cluster modules correlated with external gene information were labeled by summarizing their main functionality in red text.

In order to correlate the network modules with the health status of the elderly subjects and the immune responses induced by QIVs, the correlation coefficients between modules and traits were calculated. Six of these traits were slightly correlated with one or more modules, and the trait-module relationships with *P* ≤ 0.1 were further analyzed (**Figure 3**). Taking the trait Gender as an example, it was correlated with the Black (*cor*=0.29 *P*=0.02) and Red Module (*cor*=0.28, *P*=0.03), while the character of QIVs-induced immunity against B_Y_ vaccine strain was related to three modules, including Green (*cor*=-0.22, *P*=0.08), Black (*cor*=-0.26, *P*=0.04), and Grey (*cor*=0.24, *P*=0.06) modules. From the perspective of module-trait relationship, the Brown module was negatively correlated with General Reaction involved in the local swelling, headache, and fever (*cor*=-0.2, *P*=0.1). The Green module was positively related with the Age of the subjects (*cor*=0.25, *P*=0.05), and negatively correlated with the two traits of General Reaction (*cor*=-0.22, *P*=0.08) and QIVs-induced immunity against B_Y_ vaccine strain (*cor*=-0.22, *P*=0.08). The Black module was positively correlated with the Gender character (*cor*=0.29, *P*=0.02) and also negatively correlated with the two traits of General Reaction (*cor*=-0.19, *P*=0.1) and pre-existing immunity against B_Y_ strain (*cor*=-0.26, *P*=0.04). The Red module was positively related to the traits of Gender (*cor*=0.28, *P*=0.03) and Medical History (*cor*=0.19, *P*=0.1). The Yellow module was only positively correlated with the character of General Reaction (*cor*=0.18, *P*=0.1), while Grey module was significantly and negatively correlated with the Age of the subjects (*cor*=-0.3, *P*=0.02). These results indicate that the traits, including gender and age in the elderly, may play a key role in the immune outcomes induced by QIVs, and a set of genes from these co-expression modules may help prognose the vaccine-related adverse reaction and contribute to the persistence of protective antibody level against influenza B_Y_ vaccine virus strain.

### Module Gene Functional Enrichment Analysis

These five co-expression modules significantly related with the external gene information were used for GO and KEGG gene enrichment analysis, and the hub genes in each module were excavated based on PPI analysis. The immunological themes of these co-expressed modules were identified by GO term enrichment analysis and the top six enrichment GO terms are shown in **Table 3**. The **Brown module** was highly enriched in the biological categories involved in interleukin production, including interleukin-8, interleukin-1 beta and interleukin-6, as well as biological signal transduction, including MyD88-dependent toll-like receptor signaling pathway and the regulation of cysteine-type endopeptidase activity involved in apoptotic process. The **Green module** was highly enriched in immune responses involved in neutrophil activation, including immune response, phagocytosis, acute inflammatory response, negative regulation of mononuclear cell proliferation, as well as the components of intracellular organelles such as ficolin-1-rich granule and secretory granule lumen. The **Black module** contained the co-expressed genes related to the regulation of body fluid levels, leukocyte migration, secretory granule lumen, secretory granule lumen and SH3/SH2 adaptor activity. The **Red module** was enriched in genes related to myeloid cell homeostasis, myeloid cell differentiation, protoporphyrinogen IX metabolic process, and homeostasis of number of cells, while the **Yellow module** contained genes involved in the cellular components of myofilaments.

**Table 3:** The enrichment of genes from co-expression modules identified by WGCNA in immune-related Gene Ontology (GO) terms.

Modules (Total gene number)	Category	GO ID	Gene Number	Description of enriched GO terms (Top 6 terms)	Significance of the enrichment (*padj*)
Brown: Interleukin (951)	BP	GO:0032637	16	interleukin-8 production	2.43E-06
	BP	GO:0032611	14	interleukin-1 beta production	4.90E-05
	BP	GO:0032635	15	interleukin-6 production	5.63E-05
	BP	GO:0002755	8	MyD88-dependent toll-like receptor signaling pathway	6.54E-05
	BP	GO:0043281	21	regulation of cysteine-type endopeptidase activity involved in apoptotic process	9.90E-05
Green: Neutrophil Activation (487)	BP	GO:0002283	54	neutrophil activation involved in immune response	9.50E-15
	BP	GO:0006909	25	phagocytosis	2.34E-06
	BP	GO:0002526	16	acute inflammatory response	1.66E-05
	BP	GO:0032945	11	negative regulation of mononuclear cell proliferation	0.000144225
	CC	GO:0101002	23	ficolin-1-rich granule	3.88E-07
	CC	GO:0034774	26	secretory granule lumen	2.99E-05
Black: Fluid Regulation (163)	BP	GO:0050878	21	regulation of body fluid levels	3.54E-08
	BP	GO:0050900	14	leukocyte migration	0.000352662
	CC	GO:0034774	11	secretory granule lumen	0.001267104
	MF	GO:0045236	4	secretory granule lumen	0.001972862
	MF	GO:0005070	4	SH3/SH2 adaptor activity	0.046781552
Red: Myeloid Cell (173)	BP	GO:0002262	9	myeloid cell homeostasis	0.000973577
	BP	GO:0030099	13	myeloid cell differentiation	0.005953997
	BP	GO:0046501	3	protoporphyrinogen IX metabolic process	0.016282478
	BP	GO:0048872	9	homeostasis of number of cells	0.031503274
Yellow: Myofilament (613)	CC	GO:0036379	5	myofilament	0.005722575

One gene may be enriched in multiple terms. BP, Biological process; CC, Cellular component; MF, Molecular function; padj, P values adjusted by the multiple test correction method.

Furthermore, the potential pathways associated with QIVs-induced immunity in these five modules were investigated by KEGG enrichment analysis. The **Brown module** was enriched in genes involved in Influenza A (hsa05164, *padj*=8.23E-06), NOD-like receptor signaling pathway (hsa04621, *padj*=2.05E-05), Th17 cell differentiation (hsa04659, *padj*=0.002), Toll-like receptor signaling pathway (hsa04620, *padj*=0.002), Th1 and Th2 cell differentiation (hsa04658, *padj*= 0.0118), and Chemokine signaling pathway (hsa04062, *padj*=0.0465). The **Green module** genes were enriched in the Fc gamma R-mediated phagocytosis (hsa04666, *padj*=0.0199), while **the Black module** co-expression genes participated in Platelet activation (hsa04611, *padj*=0.0001), Complement and coagulation cascades (hsa04610, *padj*=0.0284), ECM-receptor interaction (hsa04512, *padj*=0.0284), and Chemokine signaling pathway (hsa04062, *padj*=0.0284). The pathway Mitophagy (hsa04137, *padj*=0.0088) was identified in the **Red module**, while the **Yellow module** genes were not enriched in any KEGG term.

### Identification of Hub Genes Intimately Related With the Traits of Interest

The correlation coefficients between co-expression modules and traits revealed the **Green module** with the most significant positive and negative correlation to Age and General Reaction within 7 days, in contrast to the most significant positive and negative correlation to Gender and pre-existing immunity against B_Y_ vaccine strain for the **Black module**, respectively. Therefore, these two modules were the priority for further sub-network analysis and key gene identification, and they were designated as top age-related module and top gender-related module, respectively.

The differential expression genes (DEGs) in the subjects with gender and age differences were investigated to identify the intersectional DEGs from the most trait-related gene clusters. In the top age-related module, 2 up-regulated genes were screened in the subjects ≤ 65 yrs versus those > 65 yrs. In the top gender-related module, 81 activated genes were identified in the female group versus the male group. Meanwhile, in the top age-related gene cluster, 23 DEGs were generated in subjects with vaccination-induced general reaction within 7 days versus those with no general reaction, including two up-regulated and 21 down-regulated genes. In the top gender-related gene cluster, only 10 down-regulated genes were screened in the group with seroprotection against B_Y_ strain versus the group with no seroprotection against B_Y_ strain pre-vaccination. The traits-related DEGs from these modules are shown in **Table S2**.

**Figure 4:**
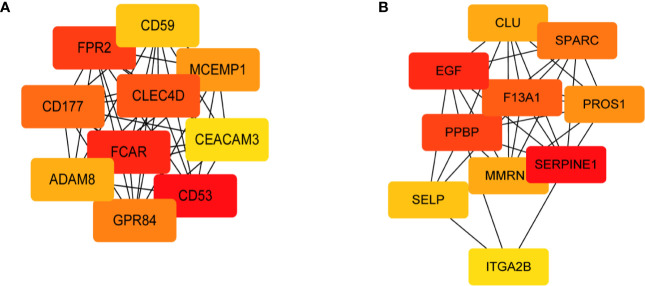
Sub-network of the top 10 hub genes extracted based on the GO terms of the two modules with a significant correlation with the QIVs-induced immunity. The nodes indicate the genes, and the edges indicate the degree of correlation. The top 10 hub genes were ranked by the MCC value, and the intensity of red color represented a higher MCC value. **(A)** Sub-network derived from the Green gene cluster; **(B)** Sub-network derived from the Black gene cluster.

PPI analysis and sub-network construction were performed for genes enriched in the GO terms relevant to vaccination-induced immunity. The sub-network was constructed using the MCL clustering method with inflation parameter set as 3, and the MCODE plugin of Cytoscape was conducted to identify the key clusters of the PPI network. By ranking the genes with the MCC value, 10 hub genes were identified in each module (**Figure 4**) and no statistical difference of MCC score occurred in the top 8 genes of each module (**Table S3**). According to the PPI analysis and gene functional annotation, *MCEMP1* and *SPARC* were deemed as the key genes with most significant relation to QIVs-induced immunity in the top age-related module and top gender-related module, respectively.

### Validation of *MCEMP1* and *SPARC* by Differential Expression Analysis of the Specific Trait Groups in the Elderly Subjects

**Figure 5:**
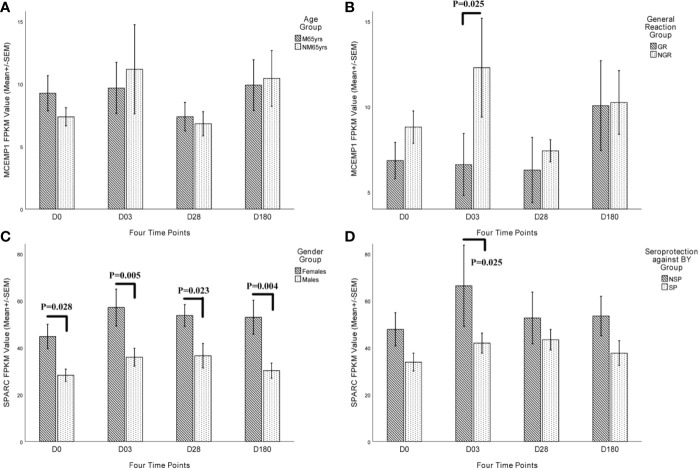
Kinetic characteristics of *MCEMP1* and *SPARC* expression in different trait groups. The differential transcriptional expression of *MCEMP1* in **(A)** different age groups and **(B)** general reaction (induced by vaccination within 7 days) groups. The differential transcriptional expression of the *SPARC* gene in **(C)** different gender groups and **(D)** seroprotection against B_Y_ influenza vaccine strain (existing immunity before vaccination) groups. Statistical significance was considered as *p*-value <0.05. FPKM, expected number of Fragments Per Kilobase of transcript sequence per Millions base pairs sequenced; M65yrs, more than 65 years old; NM65yrs, no more than 65 years old; GR, general reaction within 7 days after QIVs vaccination; NGR, no general reaction within 7 days after QIVs vaccination; NSP, no seroprotection against B_Y_ strain pre-vaccination; SP, seroprotection against B_Y_ strain pre-vaccination.

To further add weight to the key role of these two genes in the immune responses to QIVs, we analyzed the expression level of *MCEMP1* and *SPARC* in different character groups of the subjects ≥ 60 yrs. The expression of *MCEMP1* reached the peak at day 3 post-vaccination either in NM65yrs (no more than 65 years) group or M65yrs (more than 65 years) group, with higher expression level identified in NM65yrs group at day 3 and 180 after influenza immunization (**Figure 5A**). Meanwhile, the expression of *MCEMP1* was significantly higher in the no general reaction (NGR) group than in the QIVs-associated general reaction (GR) group at day 3 post-immunization (*p*=0.025) (**Figure 5B**). The other key gene *SPARC* for immune differentiation showed obviously higher expression in the female group than in the male group at four time points, reaching the maximal value at day 3 post-vaccination in the female group (*p*=0.005) and day 28 in the male groups (*p*=0.023) (**Figure 5C**). Furthermore, the *SPARC* gene showed significantly lower expression in the SP group (with seroprotection or pre-existing immunity against influenza B_Y_ vaccine strain) than in the NSP group (with no seroprotection against B_Y_ strain pre-vaccination) at day 3 (*p*=0.025) (**Figure 5D**). The differential expression analysis results demonstrated that the *MCEMP1* and *SPARC* genes could contribute to the development of QIVs-related immunity.

**Figure 6:**
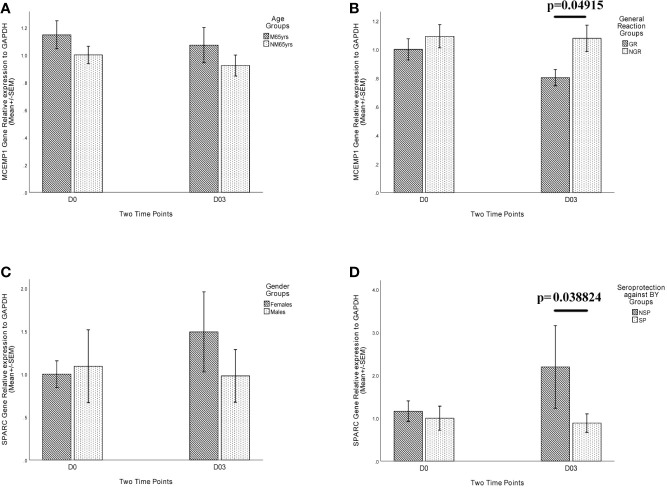
Relative expression level of *MCEMP1* and *SPARC* genes in different trait groups. The relative expression of *MCEMP1* gene among **(A)** different age groups and **(B)** general reaction (induced by vaccination within 7 days) groups. The relative expression of *SPARC* gene among **(C)** different gender groups and **(D)** seroprotection against B_Y_ influenza vaccine strain (existing immunity before vaccination) groups. The expression level of *GAPDH* was used as an internal control. Each sample was detected in triplicate. Each bar and error bar represent Mean FC (Fold Change) value and the Standard Error (SEM), respectively. Statistical significance was considered as *p*-value <0.05. FPKM, expected number of Fragments Per Kilobase of transcript sequence per Millions base pairs sequenced; M65yrs, more than 65 years old; NM65yrs, no more than 65 years old; GR, general reaction within 7 days after QIVs vaccination; NGR, no general reaction within 7 days after QIVs vaccination; NSP, no seroprotection against B_Y_ strain pre-vaccination; SP, seroprotection against B_Y_ strain pre-vaccination.

To verify the RNA-Seq results of differential expression of *MCEMP1* and *SPARC* genes among different trait groups, qRT-PCR assay was conducted, and the gene expression patterns of *MCEMP1* and *SPARC* genes were shown to be consistent with the transcriptome results in distinguishing the General Reaction Groups and Seroprotection Groups respectively (**Figure 6**). At day 3 post-vaccination, the relative expression level of *MCEMP1* gene was significantly higher in the no general reaction (NGR) group (*p*=0.04915) (**Figure 6B**), in contrast to a significantly lower relative expression level of the *SPARC* gene in the SP group (*p*=0.038244) (**Figure 6D**).

### Regulatory Roles of *MCEMP1* and *SPARC* in Cytokine Secretion (CXCL 8/IL-8, Granzyme-B)

The interferon (IFN-alpha<1.19 pg/ml, IFN-beta<1.57pg/ml, and IFN-gamma<6.46 pg/ml) and IL-10 (<1.02 pg/ml) concentrations among most plasma samples were lower than the detection limit. The dynamic expression of CCL4 and HGF cytokines could identify the peak level of vaccine-induced immune responses at day 3 post-vaccination, but could not significantly distinguish the different immune phenotypes and vaccine-related immune progression stages.

The potential immunological functions of these two genes were investigated by analyzing the regulation of the lymphocyte activity and inflammation by the genes-related immunoglobulin and/or cytokines using the plasma samples from the elderly subjects. Previous studies have shown that lower expression of *MCEMP1* enhanced the T cell activity and NK cell activity while suppressing the secretion of inflammation-induced cytokine in a mouse model with sepsis, which was identified as a type II transmembrane protein in human mast cells, exerting functionality by secreting multiple preformed inflammatory mediators (36, 37). Generally, *SPARC* is a non-structure matrix-related glycoprotein, contributing to the organization and regulation of extracellular matrix (ECM) networks, including cell shape, cell migration, composition of ECM, and molecular signal transduction (38–40), while *SPARC* not only serves as a prediction biomarker for various cancers, but also shows a significant effect on the immune cell responses, i.e., its overexpression could inhibit the migration of dendritic cells (DCs) and subsequently obstruct the development of adaptive immunity and promote inflammation (41–43). Therefore, we analyzed the relevant cytokines and categorized them as pro-inflammation (CXCL 8/IL-8) and cell-mediated immunity (Granzyme-B).

**Figure 7:**
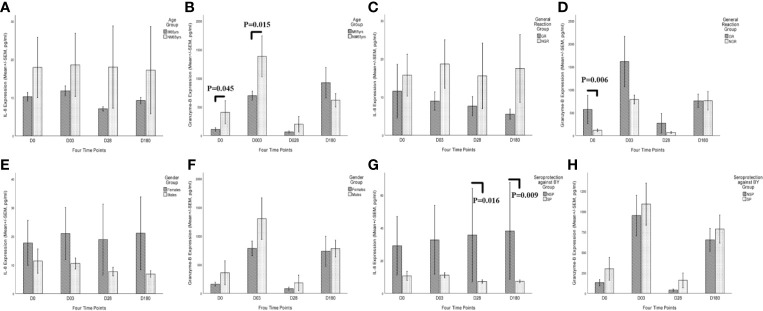
Kinetic characteristics of CXCL 8/IL-8 and Granzyme-B secretory cytokines after QIVs vaccination in the plasma in different trait groups at four time points (days 0, 3, 28, and 180). Elderly subjects with specific traits were categorized into four trait groups, including age group **(A, B)**, vaccination-related general reaction group **(C, D)**, gender group **(E, F)**, and existing seroprotection against B_Y_ group **(G, H)**. Only one subject was excluded from the cytokine response analysis due to lack of the fourth blood draw. Statistical significance was considered as *p*-value <0.05. M65yrs, more than 65 years old; NM65yrs, no more than 65 years old; GR, general reaction within 7 days after QIVs vaccination; NGR, no general reaction within 7 days after QIVs vaccinations; SP, seroprotection against B_Y_ strain pre-vaccination; NSP, no seroprotection against B_Y_ strain pre-vaccination; SP, seroprotection against B_Y_ strain pre-vaccination.

The kinetic characteristics of these two cytokines can reflect the potential progression of inflammation status and cell-mediated immunity after immunization (**Figure 7**). Consistent with previous studies, GR group showed a lower expression of *MCEMP1*, leading to the suppression of CXCL 8/IL-8 and the increased expression of Granzyme-B (**Figures 7C, D** **)**. The overexpression of *SPACR* in the female group and NSP group promoted inflammation with a high concentration of CXCL 8/IL-8 cytokine (**Figures 7E, G** **)**, while inhibiting the cell-mediated immunity with a low level of Granzyme-B (**Figures 7F, H** **)**. However, compared with the protein level, the kinetic characteristics of the mRNA expression of these two cytokine genes in the venous blood cells failed to show obvious difference in the different trait groups (age, gender, general reaction, and pre-existing immunity), and thus the cytokine concentrations rather than the mRNA expression levels were used to evaluate the immune responses, as the plasma cytokines (CXCL 8/IL-8 and Granzyme-B) are derived from a variety of cells (**Figure S2**).

## Discussion

The weaker immune responses to TIV were identified not only in infants, but also in the elderly individuals, when compared with adults (44). The efficacy of inclusion of MF59 (an oil-in-water emulsion adjuvant) in trivalent seasonal influenza vaccines was further assessed in immunological naïve infants, which showed a similar transcriptional response in infants as that in adults vaccinated with TIVs, and a positive correlation of early gene signatures of innate immunity at day 1 (including antiviral IFN genes, dendritic cell, etc.) with higher serum antibody titers at day 28 (45, 46). Previously identified biomarkers and gene signatures could only predict humoral antibody responses to influenza vaccine in young adults, but not in the elderly, due to the expression of *CXCR5* and programmed death-1 (*PD-1*) genes in circulating Tfh cells (47). Furthermore, the immune response of the elderly to influenza vaccines is highly heterogeneous, with low or no immune responses detected in some of them, which was not driven by the absence or presence of pre-existing immunity to influenza virus (48, 49). This heterogeneity highlights the necessity to investigate the transcriptional basis for the variability of immune responses to influenza vaccination in the elderly.

Safe and effective QIV vaccination is an urgent demand for the populations with a high risk of influenza infection, especially the elderly individuals ≥ 60 yrs, and the progression of vaccine-induced immunity is a network process, highlighting the importance of effector cytokines in bridging the interaction of multiple immune cells. In this study, the biological information on immune responses to QIVs was excavated based on gene co-expression patterns through WGCNA analysis of the transcriptomic data from 63 vascular blood samples. These blood samples were collected from 16 subjects at 4 time points pre- and post-immunization, excluding one sample due to lack of the fourth blood sampling. Eight co-expression modules were generated by hierarchical clustering analysis, with the cluster size ranging from 82 to 9,579 genes. Two of the eight gene clusters (Green, Black, a total of 650 genes) were significantly correlated with individual characteristics of age and gender, as well as the QIVs-induced immune responses involved in general reaction within 7 days and pre-existing immunity against B_Y_ vaccine strain.

By correlating the two gene clusters to the traits of the elderly individuals before and after influenza vaccination, the Green module was shown to be significantly positively correlated with the age of the subjects and negatively correlated with general reaction (reactogenicity). Meanwhile, the Black module was identified to have a significant positive correlation with gender dimorphism and negative correlation with pre-existing immunity against B_Y_ vaccine strain. After correlation analysis, potential hub genes were identified by GO and KEGG functional enrichment analysis. Results indicated that the genes with a similar expression pattern in the top age-related module were significantly enriched in the initial immune responses to QIVs, including phagocytosis, acute inflammatory response, negative regulation of mononuclear cell proliferation, while the genes in the top gender-related module were enriched in the cell-mediated immunity. From these two highly trait-related modules, the intersectional DEGs were excavated, with 2, 81, 23, and 10 DEGs being identified in the age, gender, general reaction, and pre-existing immunity groups, respectively. The DEGs correlated with individual characteristics were up-regulated during the immune process, while most of the DEGs related with vaccine-induced responses were down-regulated, which could be used as the potential biomarkers for the individual vaccination strategy in the elderly.

To determine the hub genes in the two modules, the genes enriched in the GO terms related to the immune responses induced by QIVs were confirmed by PPI analysis and sub-network construction. The integrated functional analysis revealed *MCEMP1* and *SPARC* as the hub genes during the development of safe and effective immunity related to influenza vaccines in the top age-related module and top gender-related module, respectively. As few studies have been performed on the association of *MCEMP1* and *SPARC* genes with influenza vaccine responses thus far, the transcriptional expression level of the two hub genes was analyzed based on the FPKM value, and the potential regulatory roles of *MCEMP1* and *SPARC* in the immunity against influenza were confirmed by analyzing the dynamic activity of the related effector cytokines CXCL 8/IL-8 and Granzyme-B.

*MCEMP1* showed a significantly higher expression in the NGR group than in the GR group, reaching the peak value at day 3 post-vaccination, highlighting the regulatory effect of dynamically expressed *MCEMP1* on the safety and tolerability to the influenza vaccines. Furthermore, the transcriptional expression level of *SPARC* was obviously higher in the female group than in the male group, reaching the peak in the former group at day 3 and in the latter group at day 28, implying higher humoral antibody and immune responses to influenza vaccines in the females (50). Intriguingly, the SP group displayed lower *SPARC* expression than NSP group, further confirming that excessive *SPARC* expression may lead to the decline of protective humoral antibody level, while *SPARC* deficiency may impair B lymphopoiesis (51, 52).

The *MCEMP1* gene is located on human chromosome 19 band p13.3, and genes adjacent to *MCEMP1* may contribute to the immune regulation of cell surface receptors, including transient expression of *CD23* molecules (IgE low-affinity receptors) (36). The *MCEMP1* gene encodes mast cell-expressed membrane protein 1 and can be expressed in mast cells, macrophages and even the brain tissue (53, 54). The effect of *MCEMP1* on QIVs-induced immunity in elderly populations remains to be elucidated, although growing studies demonstrated its diagnostic capacity on the progression of stroke (53), and the promotion effect of its high expression on inflammation and sepsis (55).

The *SPARC* gene is located on human chromosome 5 band q31-q33, and its neighboring genes contributed to the immune regulation based on the transcriptional level, such as *MFAP1* required for pre-mRNA processing, activation of T and NK cells (*IL12B* gene), as well as proliferation and differentiation of mononuclear phagocytes (*CSF1R*) (56). Human SPARC known as a secretory protein was detected among a variety of tissues, including blood, lung, and skin tissues (57). The functional role of the SPARC protein in the development of QIVs-induced immunity is being clarified, and SPARC has been the topic of various recent studies, including tumorigenesis (58), wound healing (39), lymphocytes activity (40, 59), and innate immunity (60, 61).

There are many limitations in the measurement of QIV efficacy in the elderly using only antibody titers against influenza, suggesting that cell-mediated immunity is essential for detecting anti-influenza efficacy (62). Previous studies have demonstrated the key immunological functions of the proteins encoded by the two genes. Combined with gene functional enrichment and individual characteristic analysis, we testified the immune process related to inflammation promotion and cell-mediated immunity, in which effector cytokines CXCL 8/IL-8 and Granzyme-B were selected to monitor the dynamic immune responses of inflammation and cell immunity pre- and post-immunization, respectively. Chemokine CXCL 8/IL-8 may affect vaccination as a molecular adjuvant, and the trivalent inactivated vaccine (TIV) would influence the serum cytokine level, but the live attenuated influenza vaccine (LAIV) would not (63), which was supported by the suppression of cytokine response patterns in severe pandemic 2009 H1N1 among hospitalized adults (64). In previous studies, Granzyme-B was shown as a cytolytic mediator contributing to the activation of cytotoxic T lymphocyte (CTL) and the elimination of influenza-infected cells, which can be used in diagnosis of influenza illness and influenza-induced fever (r= 1.000; *p*= 0.01) in the vaccinated elderly (65).

This is probably the first study regarding the correlation of these two genes (*MCEMP1, SPARC*) with individual traits and immune responses in the development of effective immunity against influenza after QIVs inoculation. In our study, *MCEMP1* was shown to contribute significantly to the reactogenicity within 7 day after QIVs vaccination, with higher Granzyme-B secretion and lower CXCL 8/IL-8 expression in the GR group (with vaccine associated adverse reactions) than in the NGR group (with good tolerability). Interestingly, the *SPARC* gene expression level could distinguish the females from males, which reached a peak at day 28 in the male group but at day 3 in the female group. Humoral and cell-mediated immunity showed a higher CXCL 8/IL-8 secretion and a lower Granzyme-B expression, which was consistent with previous studies reporting that females typically generate higher humoral antibody responses to seasonal influenza vaccines while the males sustain longer-lasting seroprotective antibody titers (50, 66, 67). The kinetic characteristics of the *SPARC* gene expression pattern in the NSP group were in accordance with the trait of the female group (as the NSP group mainly consisted of elderly females) and the secretion level of these two cytokines. Additionally, GO enrichment analysis indicated that MCEMP1 mainly contributed to the neutrophil-mediated immunity, including neutrophil degranulation, granulocyte activation, neutrophil activation involved in immune response, formulation of specific granules and secretory granule membranes involved in mediating the immune process. Meanwhile, SPARC was shown to participate in the immune responses to the pathogen-derived antigens (such as lipopolysaccharide and molecules of a bacterial origin) and was involved in the lumen development of cytoplasmic vesicles and secretory granules. From these results, it can be concluded that the *MCEMP1 and SPARC* genes may have an effect on the development of protective humoral and cell immunity against influenza in the elderly. These two genes could not only be used as biomarkers in the prediction of adverse reaction and immunogenicity before influenza vaccination, but also provide gender characteristics for further research to improve the QIV efficacy in the development of personalized influenza vaccines. Furthermore, due to the insufficient research on potential biomarkers for the evaluation of influenza vaccine efficacy and safety, *MCEMP1 and SPARC* genes in our study may provide a good reference for further studies.

This study has a few limitations that warrant further investigation. First, our study lacks a large sample size covering different races of older people, and thus the specificity of *MCEMP1* and *SPARC* genes could not be synthetically evaluated. Second, if the threshold concentration can significantly differentiate populations with different immune response phenotypes, then the QIVs-related reactogenicity biomarker *MCEMP1* and immune persistent biomarker *SPARC* will have greater utility. In our study cohort, the elderly enrolled demonstrated a history of influenza virus vaccination and/or infection, based on the existing immunity to four vaccine strains before inoculation, and blank samples (with non-existing immunity) should be collected to evaluate the utility of these two biomarkers (*MCEMP1* and *SPARC*) in guiding the clinical use of influenza vaccines covering a wider population. Third, the underlying mechanisms of *MCEMP1* and *SPARC* genes contributing to the development of protective immunity after influenza vaccination should have been rigorously verified in multidimensions, especially the regulation of effector cytokines (CXCL 8/IL-8, Granzyme-B). Due to the small size of the current study, our findings need to be further confirmed with a larger sample size and thus cannot be applied to different ethnic groups at present.

In this study, the hub genes related to the immunity against seasonal influenza viruses were identified by a comprehensive network-based analysis of transcriptome data and plasma cytokine responses in the elderly≥60 yrs. *MCEMP1* and *SPARC* were confirmed as the hub genes with potential influence on the outcomes of QIVs-induced immunity. The *MCEMP1* expression is negatively correlated with the QIVs-related reactogenicity within 7 day after vaccination, which would be suppressed by the CXCL 8/IL-8 and exacerbated by the Granzyme-B cytotoxic mediator. Meanwhile, the *SPARC* expression tends to increase the immune responses to the QIVs and promote the persistence of protective humoral antibody titers. These two genes can be used to predict the adverse reactions induced by QIVs, the intensity of immune responses, and the persistence of humoral antibody against influenza. This work has provided useful information for future research on the development of personalized seasonal influenza vaccines with proper immune responses and long-lasting immunity against influenza.

## Data Availability Statement

The datasets presented in this study can be found in online repositories. The names of the repository and accession number can be found here: https://www.ncbi.nlm.nih.gov/geo/, GSE151558.

## Ethics Statement

The studies involving human participants were reviewed and approved by The Clinic Institutional Ethics Review Board of Guangdong Centers for Disease Control and Prevention. The patients/participants provided their written informed consent to participate in this study.

## Author Contributions

JY, JYZ, RF, WZ, JKZ, and XY conceived the idea of this research. JY, JYZ, RF, and TH carried out the bioinformatics and data analysis. JY, JYZ, RF, WZ, and KD, XL, PZ, and JD collaborated with the interpretation of results, discussion, and review of the manuscript. JY drafted the manuscript. All authors contributed to the article and approved the submitted version.

## Conflict of Interest

JY is studying for his doctorate at Wuhan Institute of Biological Products Co., Ltd. JYZ, WZ, KD, and XL are employees in the company Wuhan Institute of Biological Products Co., Ltd. TH is studying for a master’s degree at Wuhan Institute of Biological Products Co., Ltd. XY is an employee of the company China Biotechnology Co., Ltd.

The remaining authors declare that the research was conducted in the absence of any commercial or financial relationships that could be construed as a potential conflict of interest.
