# Supplementary material for: Identifying Potential Candidate Hub Genes and Functionally Enriched Pathways in the Immune Responses to Quadrivalent Inactivated Influenza Vaccines in the Elderly Through Co-Expression Network Analysis
Source: Front Immunol. 2020 Dec 4;11:603337. doi: 10.3389/fimmu.2020.603337 (PMC7746648; doi:10.3389/fimmu.2020.603337)
Supplement: Supplementary file 1 [file Table_1.docx]

Supplementary Material

**Table S1.** The Traits of Immune Status among the 16 older adults.

| Subjects  Traits | **S04** | **S11** | **S12** | **S16** | **S25** | **S28** | **S29** | **S53** | **S07** | **S14** | **S15** | **S18** | **S26** | **S32** | **S45** | **S54** |
| --- | --- | --- | --- | --- | --- | --- | --- | --- | --- | --- | --- | --- | --- | --- | --- | --- |
| Sex | 1 | 1 | 1 | 1 | 1 | 1 | 1 | 1 | 2 | 2 | 2 | 2 | 2 | 2 | 2 | 2 |
| Age | 62 | 63 | 79 | 60 | 76 | 63 | 60 | 69 | 61 | 68 | 66 | 64 | 67 | 64 | 66 | 65 |
| Medical  History | 0 | 0 | 1 | 0 | 2 | 0 | 3 | 0 | 0 | 4 | 0 | 0 | 0 | 0 | 0 | 0 |
| Temperature | 36.2 | 36.5 | 36 | 35.6 | 36.4 | 36.2 | 36.9 | 36.1 | 37 | 36.2 | 36.5 | 35.6 | 37.2 | 35.7 | 36.2 | 36 |
| General  Reaction | 1 | 0 | 0 | 1 | 0 | 1 | 1 | 0 | 0 | 0 | 0 | 0 | 0 | 0 | 1 | 0 |
| BMI | 23.9 | 17.9 | 22.6 | 21.3 | 24.8 | 23.5 | 22.6 | 18.9 | 29.3 | 21.9 | 33.3 | 18.6 | 21.5 | 29.2 | 20.2 | 26.4 |
| BPD | 40 | 60 | 40 | 40 | 68 | 50 | 50 | 51 | 65 | 40 | 52 | 50 | 50 | 40 | 40 | 50 |
| Pre-existing Antibody against H1N1 | 80 | 10 | 5 | 80 | 5 | 20 | 5 | 5 | 640 | 10 | 20 | 80 | 40 | 20 | 5 | 10 |
| Pre-existing Antibody against  H3N2 | 5 | 20 | 40 | 40 | 5 | 10 | 40 | 80 | 20 | 5 | 10 | 80 | 40 | 10 | 40 | 5 |
| Pre-existing Antibody against  B_YAM_ | 80 | 160 | 40 | 80 | 20 | 40 | 40 | 160 | 40 | 40 | 40 | 40 | 20 | 40 | 80 | 20 |
| Pre-existing Antibody against  B_VIC_ | 40 | 160 | 20 | 40 | 40 | 10 | 40 | 20 | 5 | 10 | 5 | 20 | 5 | 20 | 20 | 5 |

Table S2. Differential Expression Genes (DEGs) Identified from the Trait-related Modules.

|  | Top age-related module | Top gender-related module |
| --- | --- | --- |
| Age | **IL1B, USP18** | / |
| Gender | / | **NRGN, PPBP, MT-TM, F13A1, TUBB1, SPARC, TREML1, GP9, MT-TQ, ITGA2B, CLU, PF4V1, PRKAR2B, CA2, CXCR2P1, MPIG6B, VSIG2, MMD, CXCL5, C2orf88, GAS2L1, ITGB3, ESAM, GFI1B, ITGB5, GP1BA, SELP, CTTN, ABLIM3, Z82206.1, FHL1, CTDSPL, AP001189.3, PEAR1, FO538757.2, GP6, ELOVL7, LY6G6E, TSPAN9, TBXA2R, MFAP3L, JAM3, LTBP1, RHOBTB1, SLC24A3, RAB6B, MYLK, PCSK6, RGS6, SEC14L5, EGF, F2RL3, VIL1, ARHGAP6, SAMD14, FSTL1, MEIS1, TGFB1I1, TTC7B, CXCL3, PRTFDC1, PCYT1B, PARD3, TRAPPC3L, MYCT1, C1orf116, VEPH1, AL121983.2, HRK, CNN1, PLXNB3, SLC6A4, PTPRF, PKHD1L1, EHD2, LEFTY1, LIPH, TRIM40, WASF3, MMP1, GLYATL2** |
| General Reaction | **AK5, CCR12P,** FCGR3B, IFITM3, FFAR2, PROK2, IFIT3, IRF7, IL1RN, IFIT1, CSRNP1, KRT23, TNFAIP6, PLSCR1, RSAD2, IFI44, C3orf86, SLC22A4, CYBRD1, ASPRV1, ETV7, AL121760.1, SMPDL3B | / |
| B_Y_ | / | F13A1, CPNE5, GGTA1P, IGFBP2, PROS1, SAMD14, FSTL1, TRAPPC3L, AL121983.2, HOXA10 |

Note: The DEGs in the table with bold font were up-regulated, while the other DEGs were down-regulated.

Table S3. MCC Values of Top 10 Genes in Network string interactions.tsv ranked by MCC method in Black and Green Modules.

| Green Module | | | Black Module | | |
| --- | --- | --- | --- | --- | --- |
| Rank | Name | Score | Rank | Name | Score |
| 1 | CD53 | 8.72E+10 | 1 | SERPINE1 | 5130 |
| 2 | FCAR | 8.72E+10 | 2 | EGF | 5108 |
| 3 | FPR2 | 8.72E+10 | 3 | PPBP | 5106 |
| 4 | CLEC4D | 8.72E+10 | 4 | F13A1 | 5064 |
| 5 | CD177 | 8.72E+10 | 5 | SPARC | 5046 |
| 6 | GPR84 | 8.72E+10 | 6 | PROS1 | 5041 |
| 7 | MCEMP1 | 8.72E+10 | 7 | CLU | 5040 |
| 8 | ADAM8 | 8.72E+10 | 8 | MMRN1 | 5040 |
| 9 | CD59 | 8.72E+10 | 9 | SELP | 254 |
| 10 | CEACAM3 | 8.72E+10 | 10 | ITGA2B | 199 |


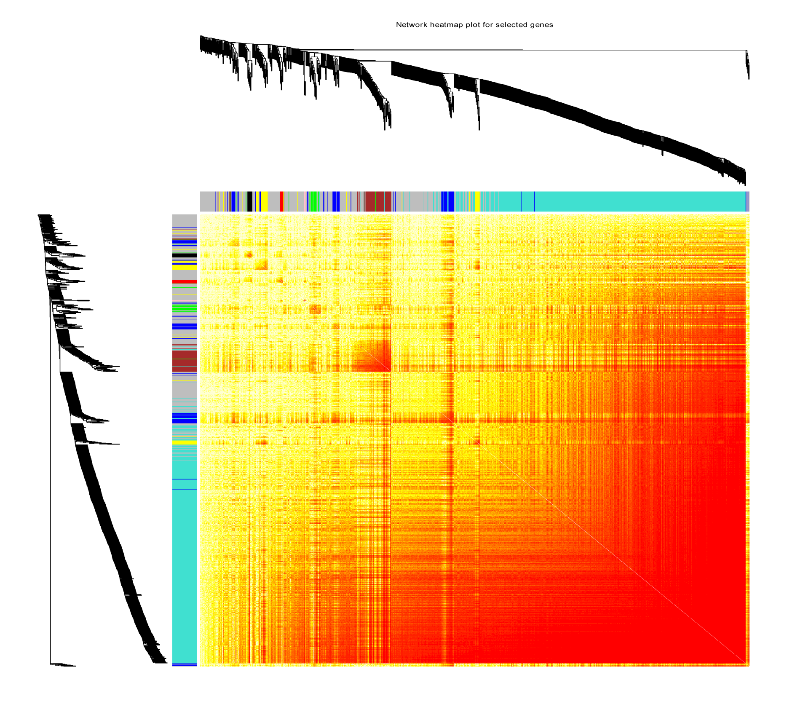


Figure S1. Heatmap of module adjacency. The red area mainly presented along the diagonal, and the intensity of the red color represented high correlation among the pairwise genes.


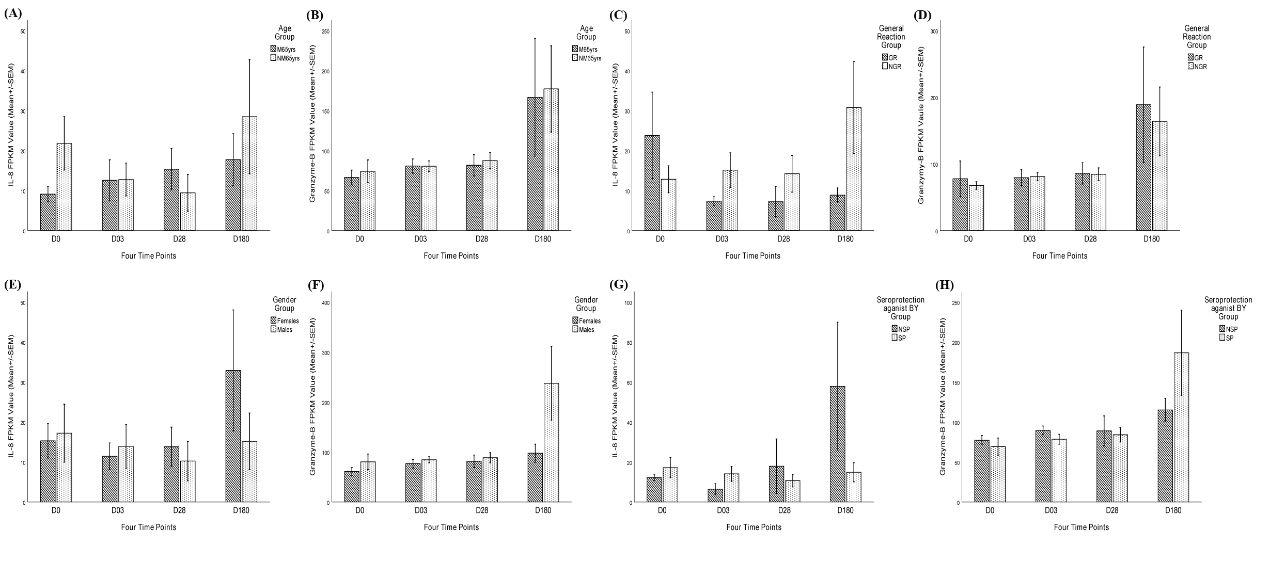


Figure S2. Kinetic characteristics of *CXCL 8/IL-8* and *Granzyme-B* mRNA expression after QIVs vaccination in the venous whole blood among different trait groups at four time points (day 0, 3, 28, and 180). Elderly subjects with specific traits were categorized into four trait groups, including age group (A, B), vaccination-related general reaction group (C, D), gender group (E, F), and existing seroprotection against B_Y_ group (G, H). Only one subject was excluded from the cytokine response analysis due to lack of the fourth blood draw. Abbreviations: M65yrs=more than 65 years old; NM65yrs=no more than 65 years old; GR=general reaction after QIVs vaccination within 7 days; NGR=no general reaction after QIVs vaccination within 7 days; SP=seroprotection against B_Y_ strain pre-vaccination; NSP= no seroprotection against B_Y_ strain pre-vaccination; SP=seroprotection against B_Y_ strain pre-vaccination.
